# Dr. Leigh H. Hunt

**Published:** 1883-05

**Authors:** 


### DR. LEIGH H. HUNT.

It was with sincere sorrow that the resignation of the medical editor of this journal was received. He had exhibited such unusual editorial ability, and had proved so genial and kindly in his editorial relations, that it was a severe blow to all his associates.

But inexorable duty demanded his withdrawal, a duty so plain and uncompromising that there was nothing left but acquiescence. He has the very best wishes of The Independent Practitioner for his future welfare, but his departure throws a quadrupled task upon his unfortunate associate, and this must account for the lack of medical matter in this number. Next month we hope to do better.

